# MeCP2 haplodeficiency and early-life stress interaction on anxiety-like behavior in adolescent female mice

**DOI:** 10.1186/s11689-021-09409-7

**Published:** 2021-12-11

**Authors:** María Abellán-Álvaro, Oliver Stork, Carmen Agustín-Pavón, Mónica Santos

**Affiliations:** 1 Unitat Mixta d’Investigació en Neuroanatomia Funcional, Departamento de Biologia Cel·lular, Biologia Funcional i Antropologia Física, Universitat de València, 46100 Burjassot, València, Spain; 2 Department of Genetics and Molecular Neurobiology, Institute of Biology, Otto-von-Guericke University, 39120 Magdeburg, Germany; 3 CNC–Center for Neuroscience and Cell Biology, University of Coimbra, 3004-504 Coimbra, Portugal

**Keywords:** Maternal separation, Rett syndrome, c-FOS, Arginine-vasopressin, Corticotropin-releasing hormone

## Abstract

**Background:**

Early-life stress can leave persistent epigenetic marks that may modulate vulnerability to psychiatric conditions later in life, including anxiety, depression and stress-related disorders. These are complex disorders with both environmental and genetic influences contributing to their etiology. Methyl-CpG Binding Protein 2 (MeCP2) has been attributed a key role in the control of neuronal activity-dependent gene expression and is a master regulator of experience-dependent epigenetic programming. Moreover, mutations in the *MECP2* gene are the primary cause of Rett syndrome and, to a lesser extent, of a range of other major neurodevelopmental disorders. Here, we aim to study the interaction of MeCP2 with early-life stress in variables known to be affected by this environmental manipulation, namely anxiety-like behavior and activity of the underlying neural circuits.

**Methods:**

Using *Mecp2* heterozygous and wild-type female mice we investigated the effects of the interaction of *Mecp2* haplodeficiency with maternal separation later in life, by assessing anxiety-related behaviors and measuring concomitant c-FOS expression in stress- and anxiety-related brain regions of adolescent females. Moreover, arginine vasopressin and corticotropin-releasing hormone neurons of the paraventricular hypothalamic nucleus were analyzed for neuronal activation.

**Results:**

In wild-type mice, maternal separation caused a reduction in anxiety-like behavior and in the activation of the hypothalamic paraventricular nucleus, specifically in corticotropin-releasing hormone-positive cells, after the elevated plus maze. This effect of maternal separation was not observed in *Mecp2* heterozygous females that per se show decreased anxiety-like behavior and concomitant decreased paraventricular nuclei activation.

**Conclusions:**

Our data supports that MeCP2 is an essential component of HPA axis reprogramming and underlies the differential response to anxiogenic situations later in life.

## Introduction

Early-life stress (ELS) constitutes a major risk factor for the development of psychiatric disorders, including anxiety disorders. These disorders are the most frequent mental disorders and impose an enormous societal and economic burden [1, 2]. Here, we address the potential involvement of decreased functionality of the X-linked methyl-CpG binding protein 2 (MeCP2), in mediating ELS effects on susceptibility to the development of abnormal anxious responses.

MeCP2 is a multifaceted protein with roles in transcriptional regulation, epigenetic programs, alternative splicing, microRNA processing and chromatin remodeling, thereby facilitating several biological processes [3]. In particular, MeCP2 has been attributed a key role in the control of neuronal activity-dependent gene expression [4, 5] and in experience-dependent epigenetic programming [6, 7]. As an epigenetic reader, MeCP2 controls critical periods of postnatal development, which are periods of high plasticity and sensitivity to the environment [8].

The activity of the hypothalamic-pituitary-adrenal (HPA) axis is characterized by a prominent critical period of plasticity [9]. Adverse events occurring during this period are engraved in ‘permanent’ epigenetic marks, with MeCP2 being a master regulator of these processes [6]. This way, early life adversity will have an impact in the stress response later in life, when individuals are faced with a stressor [9]. Moreover, MeCP2 is known to regulate the expression of corticotropin-releasing hormone (CRH) and arginine vasopressin (AVP) [6, 10], factors that ultimately control the secretion of corticosteroids into the bloodstream upon stress.

Loss-of-function mutations in the *MECP2* gene are the major cause of Rett syndrome (RTT), a neurodevelopmental disorder that primarily affects females [11]. In its classical presentation, RTT patients show motor impairments, breathing abnormalities, loss of speech, intellectual disability, repetitive behaviors, and autistic features [12]. Males usually have a severe and fatal neonatal encephalopathy (reviewed in [13]. To a lesser extent, *MECP2* mutations are also responsible by other major neurodevelopmental disorders such as X-linked mental retardation, autism, Angelman syndrome or schizophrenia [14]. By contrast, individuals with the *MECP2* duplication syndrome (MDS), that affects mainly males, show intellectual disability and autism [15]. The involvement of MeCP2 in multiple neurodevelopmental disorders demonstrates that it occupies a central role in the postnatal development of the brain.

Importantly, RTT patients show a blunted decrease of around 50% of cortisol concentration from morning to evening, as compared with typical values of 90% decrease in healthy individuals [16]. Interestingly, RTT cortisol patterns are significantly related to mutation severity. Conversely, MDS patients showed an atypical diurnal cortisol response, with cortisol levels failing to show the typical increase after waking, and, again, altered cortisol patterns were associated with clinical severity [17].

Several different genetic mouse models have been generated that mimic the major human behavioral, neuroanatomical, and biochemical aspects of the different disorders (reviewed in [18]), allowing the study of the underlying mechanisms. Mouse models of RTT show an altered corticosterone response to stress [10, 19–21] suggesting dysregulation of the HPA axis and an abnormal stress response. Indeed, *Crh* gene is a direct target of MeCP2 [22, 23] and mouse models of RTT show dysregulated levels of *Crh* [10, 21, 22, 24].

In this scenario, it is plausible that decreased functionality of MeCP2 early in life, as that seen in RTT patients, will impact the epigenetic programming of the HPA axis thereby affecting their vulnerability to develop anxiety and stress-related disorders later in life, when encountering a stressor. In fact, anxiety-like behavior is a prominent component of RTT’s behavioral phenotype [25]. Whereas girls with RTT display altered anxious behavior, female mice model of RTT show reduced reactivity in anxiogenic tests such as the elevated plus maze [26, 27]. Moreover, in boys with MDS anxiety is co-morbid [28] and transgenic mice that overexpress two to three-fold normal MeCP2 levels show heightened anxiety-like behavior [26]. On the one hand, dysregulation of MeCP2 levels and/or function can modulate susceptibility to develop anxiety and stress-related disorders in *MECP2*-related disorders. On the other hand, *MECP2* gene can itself constitute a genetic risk factor for the development of anxiety and stress-related disorders, though future studies are needed to investigate this later possibility.

To our knowledge, the effects of ELS in anxious behavior in *Mecp2*-deficient mice have not been experimentally tested. In this study, we used *Mecp2* heterozygous (*Mecp2*.het) and wild-type (WT) female mice that were, or were not, submitted to maternal separation (MS), a classical rodent paradigm to study ELS [29, 30]. Interfering at early developmental timepoints can affect the programming of the HPA axis with consequences in emotional behavior and neuronal functionality later in life [29, 31]. Therefore, young adult mice were tested in the elevated plus maze (EPM) and open field (OF) to measure anxiety-like behaviors and in the forced swimming test (FST) to evaluate depression-like behavior. After a second exposure to the EPM (in bright, aversive conditions), animals were sacrificed and FOS proto-oncogene (c-FOS) immunohistochemistry was used to analyze neuronal activation in anxiety- and stress-related brain regions [32]. Finally, we checked whether c-FOS co-localization with either CRH and AVP in the paraventricular hypothalamic nucleus (Pa) was affected by MS and MeCP2 functionality.

## Material and methods

### Animals

For this study, we used the *Mecp2*.het females and their wild-type littermates from our colony. To establish the colony, we purchased *Mecp2*.het females from Jackson Laboratory (stock #003890, B6.129P2(C)-Mecp2^tm1.1Bird/J^) [33] and maintained breeding pairs at our animal facility, by crossing these females with C57Bl/6J WT males. Experimental females were weaned at postnatal day (PND) 23 and housed in groups of 2–5 animals in standard laboratory cages with controlled humidity and temperature (22 °C), a 12:12-h light/dark cycle, and water and food available ad libitum. Genotyping was performed with DNA extracted from ear biopsies according to the protocol supplied by the Jackson Laboratory for this strain. Behavioral testing was performed during the dark phase of the animals.

All the procedures were carried out in strict accordance with the EU directive 2010/63/EU. The protocols were approved by the Ethics in Animal Experimentation Committee of the University of Valencia.

### Early-life stress (ELS)

To induce ELS we used the MS protocol. From PND3 to PND21 pups (WT-MS, *n* = 13; *Mecp2*.het-MS, *n* = 18) were separated from the dam as a group, and kept in a new cage filled with sawdust and warming red light for 3 h per day, after which pups were returned to their home cage. Control animals were included (WT-naive, *n* = 14; *Mecp2*.het-naive, *n* = 12) that were maintained undisturbed with their dams in the home cage until weaning. All these animals were subjected to the battery of behavioral tests.

### Open field (OF)

Animals (WT-naive, *n* = 14; WT-MS, *n* = 13; *Mecp2*.het-naive, *n* = 12; *Mecp2*.het-MS, *n* = 18) were allowed to explore a squared arena (50 cm × 50 cm × 50 cm) in a 20-min session, under red light illumination. The total distance travelled was used a measure of activity and the distance travelled and time spent in a predefined center area (25 cm × 25 cm) versus the rest of the arena was used to assess anxiety-like behavior. Movement of mice was tracked using an automated tracking system (ANY-maze^TM^ Video Tracking System, Stoelting Co., Wood Dale, IL, USA).

### Forced swimming test (FST)

To assess depressive-like state mice were tested in the FST. Mice (WT-naive, *n* = 14; WT-MS, *n* = 13; *Mecp2*.het-naive, *n* = 12; *Mecp2*.het-MS, *n* = 18) were put in an inescapable transparent jar filled with water and allowed to swim for a 5-min period. The time animals spent immobile was measured. Mice were dried out in paper towel before they were returned to their home cage.

### Elevated plus maze (EPM)

Mice were tested in the EPM in two sessions (WT-naive, *n* = 14; WT-MS, *n* = 13; *Mecp2*.het-naive, *n* = 12; *Mecp2*.het-MS, *n* = 18). The first EPM session was the first test of the behavioral battery and was performed under red light conditions. The second EPM session was the last test of the battery and was performed under bright light, aversive conditions, and 1 h after the test animals were sacrificed. The maze consisted of two opposing closed arms (in cm, 30 length × 6 wide × 15 height) and two opposing open arms (in cm, 30 length × 6 wide) extending from a common central region (6 × 6 cm) to form a ‘plus’ shape. The arms were elevated 40 cm above the floor. In a single 5-min trial, animals were placed in the center of the maze and the time spent, distance travelled and number of entries in the open and closed arms were recorded. Movement of mice was tracked using an automated tracking system (ANY-maze^TM^ Video Tracking System, Stoelting Co.).

### Histology

A random subset of the animals used in the behavioral battery was selected for anatomical studies. Animals (WT-naïve, *n* = 4; *Mecp2*.het-naïve, *n* = 5; WT-MS, *n* = 7; *Mecp2*.het-MS, *n* = 6) were deeply anaesthetized 1 h after the last EPM session (bright conditions), using a mixture of ketamine (75 mg/kg) and medetomidine (1 mg/kg) and transcardially perfused with saline solution followed by 4% paraformaldehyde in 0.1 M phosphate buffer (PB, pH 7.4). Brains were carefully removed from the skull, post-fixed in the same fixative for 4 h and placed into a 30% sucrose solution in 0.1 M phosphate buffered saline (PBS, pH 7.6) until they sank. The brains were then frozen and cut in six series of 40-μm-thick coronal sections with a freezing microtome. Free-floating sections were frozen in 30% sucrose in PB/0.02% sodium azide (0.1 M pH 7.4) for their posterior processing.

### c-FOS immunohistochemistry

One out of six brain slices parallel series of each subject was processed for the immunohistochemical detection of c-FOS. To do so, endogenous peroxidase was inactivated with 1% H_2_O_2_ in tris-buffered saline (TBS) (0.05M, pH 7.6) for 30 min at room temperature (RT). Next, sections were incubated in blocking solution containing 3% normal goat serum (NGS) in TBS-triton X100 (Tx) 0.3% for 1 h at RT. Then, sections were incubated in rabbit anti-c-FOS (1:10000, sc-52, Santa Cruz Biotechnology, Santa Cruz, CA, USA) in TBS-Tx with 3% NGS 24 h at 4 °C, followed by an incubation with biotinylated goat anti-rabbit IgG (1:200, BA1000, Vector Laboratories, Peterborough, UK) in TBS-Tx with 2% NGS for 2 h at RT. Afterwards, sections were incubated in ABC Elite (1:50, PK-6100, Vector Laboratories) in TBS-Tx for 90 min at RT. Finally, the resulting peroxidase labelling was revealed with 0.025% 3-3′-diaminobenzidine (DAB, Sigma, St. Louis, MO, USA) in PB (0.1 M, pH 8.0) and 0.01% H_2_O_2_ for 25 min. Sections were mounted onto gelatinized slides, dehydrated in alcohols, cleared with xylene and cover slipped with Entellan (Merck Millipore, Burlington, MA, USA).

### c-FOS/AVP and c-FOS/CRH double immunofluorescence

We obtained double immunofluorescent labelling for c-FOS/AVP and c-FOS/CRH in two out of six brain slices parallel series of each subject. Brain slices were first incubated with 1% sodium borohydride in 0.05 M TBS for 30 min at RT to block the endogenous fluorescence of the tissue. Next, sections were incubated in a blocking solution containing 3% normal donkey serum (NDS) in TBS-Tx 0.3% for 1 h at RT. Afterwards, each series was incubated for 48 h at 4 °C with a mixture of primary antibodies containing rabbit anti-AVP (1:1000, AB1565, Sigma-Aldrich) or rabbit anti-CRH (1:500, C5348, Sigma-Aldrich) and goat anti-c-FOS (1:500, sc-52, Santa Cruz Biotechnology, Inc.) in TBS-Tx 0.3%/ NDS 2%. Next, brain slices were incubated for 90 min at RT with appropriate secondary antibodies Rhodamine Red-X Donkey anti-Rabbit (1:200, 711-295-152, Jackson ImmunoResearch) and Alexa Fluor® 488 Donkey Anti-Goat (1:200, A-11055 Invitrogen) in TBS-Tx0.3% with 2% NDS. Finally, sections were mounted onto gelatinized slides and cover-slipped with fluorescence mounting medium (Fluoromount, Merck).

### Image acquisition, processing, and mapping

After c-FOS immunohistochemistry, we quantified c-FOS-positive nuclei in representative levels (according to the stereotaxic atlas of Paxinos & Franklin [34] of the bed nucleus of the stria terminalis lateral division, dorsal part (BSTLD; Bregma 0.38/0.02 mm), the ventral part of the lateral septum (LSV; Bregma 0.62/− 0.10 mm), the paraventricular hypothalamic nucleus (Pa; Bregma − 0.7/− 1.06 mm), the paraventricular thalamic nucleus (PV; Bregma − 0.94/− 2.18 mm) and the dentate gyrus (DG; Bregma − 1.34/− 2.92 mm). In addition, neuroanatomical references, as the anterior commissure, were used to reduce variability between the photomicrographs. Photomicrographs of these frames were obtained in both hemispheres (when possible) using a digital camera (Leica DFC495, Wetzlar, Germany) attached to a microscope Leitz DMRB (Leica AG, Wetzlar, Germany) with a × 20 objective (counting frame of 441 × 330 μm) in the case of BSTLD and LSV, and in the case of Pa, PV and DG they were photomicrographed with a × 10 objective (counting frame of 881 × 661 μm). After that, positive cells of each photomicrograph were counted manually using Fiji [35] cell counter plugin by an experimenter blind to the condition of the animals.

After c-FOS/AVP and c-FOS/CRH double immunofluorescence, photomicrographs of both hemispheres at the level of Pa (Bregma − 0.7/− 1.06 mm) were obtained using Leica TCS SPE confocal microscope. Then, an experimenter blind to the condition of the animals counted the number of c-FOS-positive nuclei using the ImageJ Cell Counter analysis tool and co-expression of these nuclei with either AVP or CRH was verified visually and the number of double immunoreactive cells registered.

### Statistical analysis

Behavioral and histological data were analyzed with two-way ANOVA with treatment (naïve or MS) and genotype (WT or *Mecp2*.het) as between-subject sources of variance, followed by post hoc Bonferroni correction for multiple tests. Significance was set at *p* < 0.05. Data were analyzed using the software IBM SPSS Statistics 22.0.

## Results

WT and *Mecp2*.het adolescent females (age 5 to 6 weeks old), naive and MS groups, were assessed for anxiety-like and depressive-like states to study the effects of *Mecp2* and ELS interaction of in the vulnerability of animals to adversity later in life. Thus, experimental subjects were submitted to tests starting with the EPM, OF, FST, and finishing with a second EPM performed in bright light conditions, 1 h after which animals were sacrificed to assess c-FOS activation patterns.

### MS renders female mice more resilient to anxiety- and depressive-like behaviors, an effect recapitulated by MeCP2 deficiency

#### Elevated plus maze

**Fig. 1:**
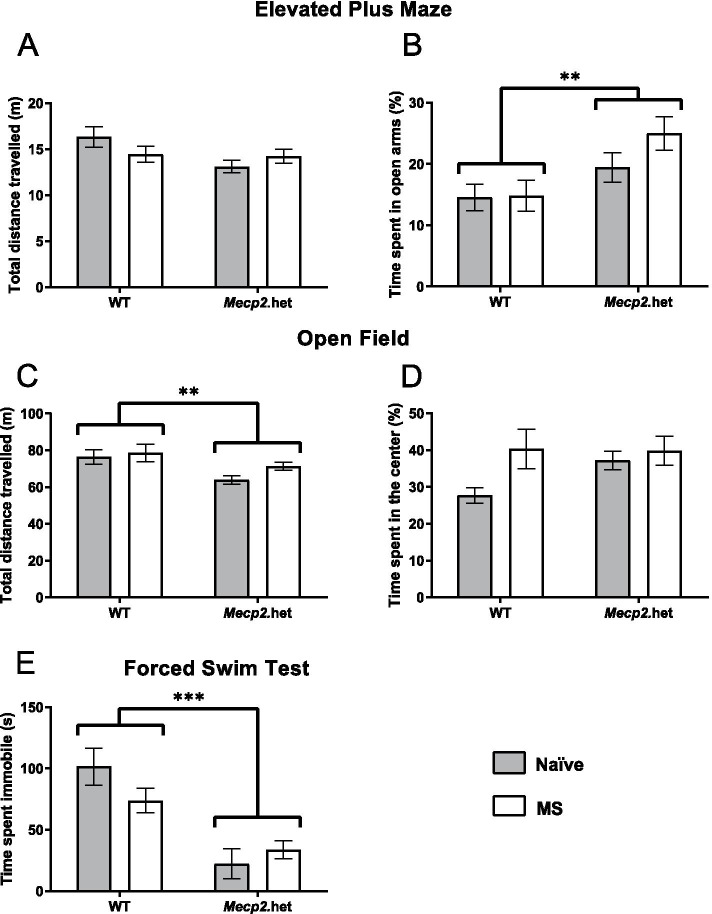
Young adult *Mecp2* heterozygous females show reduced anxiety-like and depressive-like behaviors. WT and *Mecp2*.het females, both naive and MS groups, were tested in the EPM, OF, and FST to assess anxiety-like and depressive-like behaviors (WT-naive, *n* = 14; WT-MS, *n* = 13; *Mecp2*.het-naive, *n* = 12; *Mecp2*.het-MS, *n* = 18). In the EPM, **A** no differences were found among groups in the total distance travelled in the maze, but **B** *Mecp2*.het females spent significantly longer time in the open arms of the maze as compared to WT littermates. In the open field, **C** *Mecp2*.het females showed a reduction in the total distance traveled in the maze, as compared to WT animals, but **D** no differences were found in the percentage of time spent in center among groups. In the FST, **E** *Mecp2*.het females spent significantly less time immobile than their WT littermate controls. EPM, elevated plus maze; FST, forced swim test; *Mecp2*.het, *Mecp2* heterozygous females; MS, maternal separation; OF, open field; WT, wild type; vs, *versus*. **p < 0.01, ***p < 0.001

Animals were first tested in the EPM (under red light conditions) in a single 5-min session. No differences were observed among groups in the total distance travelled in the maze (Fig. 1A). However, two-way ANOVA revealed a statistically significant genotype effect (*F*_(1, 53)_ = 8.633, *p* = 0.0049) in which *Mecp2*.het females spent more time in the open arms than WT animals (Fig. 1B), revealing a decreased anxiety-like phenotype in *Mecp2*.het females. We could not observe here an effect of the MS, as statistical analysis revealed no significant differences (*p* > 0.5; Fig. 1B).

#### Open field

Spontaneous locomotor activity and exploration were measured in the OF apparatus in a 20-min session. When analyzing the total distance travelled in the maze, two-way ANOVA showed a statistically significant genotype effect (*F*_(1, 53)_ = 8.461, *p* = 0.0053; Fig. 1C) with *Mecp2*.het females travelling a smaller total distance than their WT controls. Regarding time spent in the center of the apparatus in relation to the remaining area, we did not find a statistically significant effect of any factor (*p* > 0.05; Fig. 1D).

#### Forced swimming test

The performance of WT and *Mecp2*.het females was evaluated in the FST by measuring the immobility time in a 5-min session. Two-way ANOVA revealed a statistically significant genotype effect (*F*_(1, 51)_ = 27.09 *p* < 0.0001; Fig. 1E) showing that *Mecp2*.het females have lower immobility times than WT animals, indicative of an antidepressant-like phenotype. In addition, we also observed a trend towards significance in group x genotype interaction effect (*F*_(1, 51)_ = 3.023 *p* = 0.0881; Fig. 1E). Post hoc comparisons showed that naïve *Mecp2*.het females have lower immobility times than naïve WT animals (WT-naive vs. *Mecp2*.het-naïve, *t*_(51)_ = 4.757 *p* < 0.0001). In the MS groups, no statistically significant differences in immobility times were detected between WT and *Mecp2*.het females (WT-MS vs. *Mecp2*.het-MS, *t*_(51)_ = 2.625 *p* = 0.0684; Fig. 1E). This data suggests that MS induces a change in depressive-like behavior in WT females, and that deficiency of MeCP2 in the *Mecp2*.het females per se occludes this phenotype. Although we do not observe an effect of MS in *Mecp2*.het females, we cannot exclude that it can be masked by a floor effect given the already very low immobility time in the *Mecp2*-naive group.

#### Elevated plus maze-bright

**Fig. 2:**
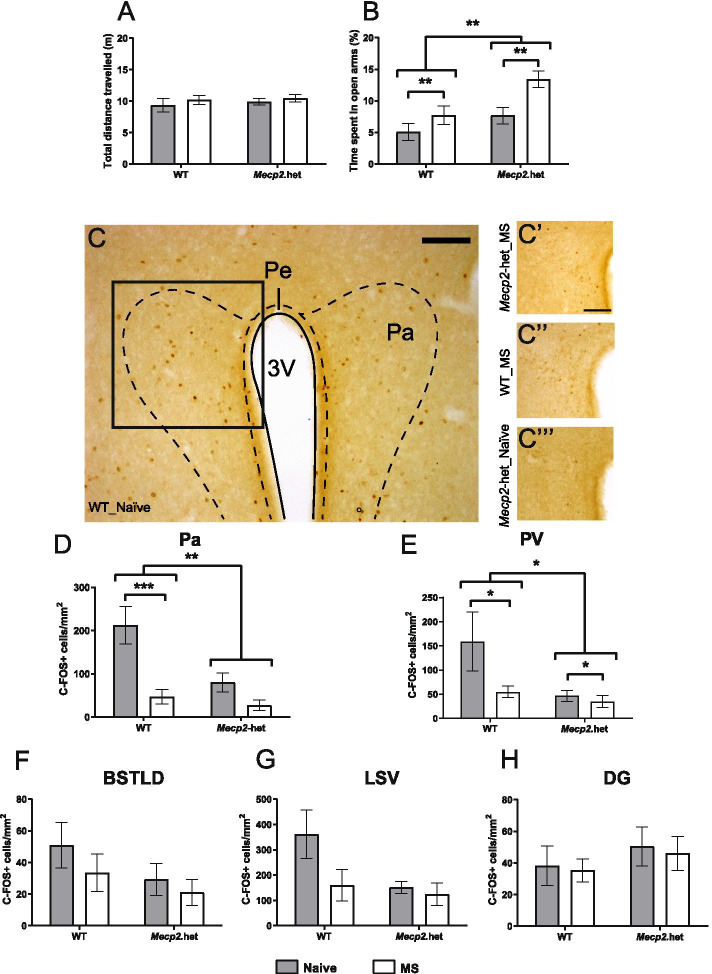
*Mecp2* haplodeficiency and maternal separation reduced anxiety-like behavior and neuronal activation in the paraventricular nuclei upon stress. WT and *Mecp2*.het females, both naive and MS animals, were re-exposed to the EPM now under bright light conditions, a more aversive version (WT-naive, *n* = 14; WT-MS, *n* = 13; *Mecp2*.het-naive, *n* = 12; *Mecp2*.het-MS, *n* = 18). **A** No differences were found among groups in the total distance travelled in the maze. **B** *Mecp2*.het females spent significantly longer time in the open arms of the maze, as compared to WT littermates, an effect further increased by MS. One hour after behavioral testing in the aversive EPM, a subset of the animals was sacrificed for c-FOS immunohistochemistry and quantification (WT-naïve, *n* = 4; WT-MS, *n* = 7; *Mecp2*.het-naïve, *n* = 5; *Mecp2*.het-MS, *n* = 6). **C** Representative photomicrographs of c-FOS staining in the paraventricular hypothalamic nucleus of WT-naive, **C’** Mecp2.het-MS, **C”** WT-MS, and **C”’** Mecp2.het-naive. Quantification of c-FOS-positive neurons in WT and *Mecp2*.het females, both naive and MS groups, in **D** Pa, **E** PV, **F** BSTLD, **G** LSV, and **H** DG. Pa, paraventricular hypothalamic nucleus; PV, paraventricular thalamic nucleus, BSTLD, bed nucleus of stria terminalis lateral division, dorsal part; LSV, lateral septum, ventral part; DG, dentate gyrus of the hippocampus; EPM, elevated plus maze; *Mecp2*.het, *Mecp2* heterozygous females; MS, maternal separation; WT, wild type; vs, *versus*. *p < 0.05, **p < 0.01, ***p < 0.001

Finally, all animals were tested in a more anxiogenic version of the EPM, under bright light conditions. Here, no differences were observed in the total distance travelled among groups (*p* > 0.5; Fig. 2A), showing that in this environment general activity is not affected by either genotype or prior stress experience. However, when we analyzed the percentage of time spent in the open arms of the maze, two-way ANOVA confirmed the genotype effect clearly seen in the previous tests, and further revealed a MS group effect. Specifically, *Mecp2*.het females show reduced anxiety levels, as revealed by the significantly higher percentage of time spent in the open arms when compared with WT animals (genotype effect *F*_(1, 53)_ = 9.095; *p* = 0.0039). Additionally, MS significantly increased the percentage of time spent in the open arms as compared with naïve animals (group effect *F*_(1, 53)_ = 9.304; *p* = 0.0036; Fig. 2B). Thus, WT and *Mecp2*.het females exposed to MS show lower levels of anxiety in face of an anxiogenic situation.

### MS and *Mecp2* deficiency decrease neuronal activation in the paraventricular nuclei upon exposure to a stressor

Mice were sacrificed 1 h after exposure to the bright EPM to allow for subsequent quantification of c-FOS-positive nuclei in brain regions (i) known to be activated after EPM exposure and (ii) related to stress and anxiety processing [32]. In the Pa, statistical analysis revealed a significant genotype x group interaction effect (Fig. 2C, D; *F*_(1, 16)_ = 5.801 *p* = 0.028). Post hoc tests showed that MS reduced the number of c-FOS-positive nuclei in WT animals (Bonferroni correction; *F*_(1, 16)_ = 24.453 *p* < 0.001). In addition, *Mecp2* deficiency resulted in a strong reduction of c-FOS-positive nuclei, as that seen in the *Mecp2*.het-naive females, when compared to WT-naive animals (*F*_(1, 16)_ = 14.520 *p* = 0.002) and MS did not significantly add up to the effect (*p* > 0.5).

Similarly, in the PV, we found a significant effect of both group (*F*_(1, 18)_ = 5.382 *p* = 0.032) and genotype (*F*_(1, 18)_ = 6.990 *p* = 0.017). Overall, MS resulted in a reduction of c-FOS-positive nuclei, particularly noticed in WT animals, and *Mecp2*.het females had a lower number of c-FOS-positive nuclei than WT animals (Fig. 2E). In these two brain regions, we cannot exclude that the lack of effect of MS in the number of c-FOS-positive nuclei observed in *Mecp2*.het females is masked by the fact that naive *Mecp2*.het females have already very low numbers of c-FOS-positive cells.

In the BSTLD and LSV, regions known to be activated by EPM, we did not observe statistically significant differences in the number of c-FOS-positive nuclei between genotypes or groups, though the tendency towards low density of c-FOS nuclei in WT-MS and *Mecp2*.het females was similar to that found in Pa and PV (*p* > 0.5; Fig. 2F, G).

Finally, no significant differences were found in the DG with respect to genotype or group (*p* > 0.5; Fig. 2H).

### MS and *Mecp2* deficiency decrease the density of double-labeled c-FOS/CRH neurons in the Pa

**Fig. 3:**
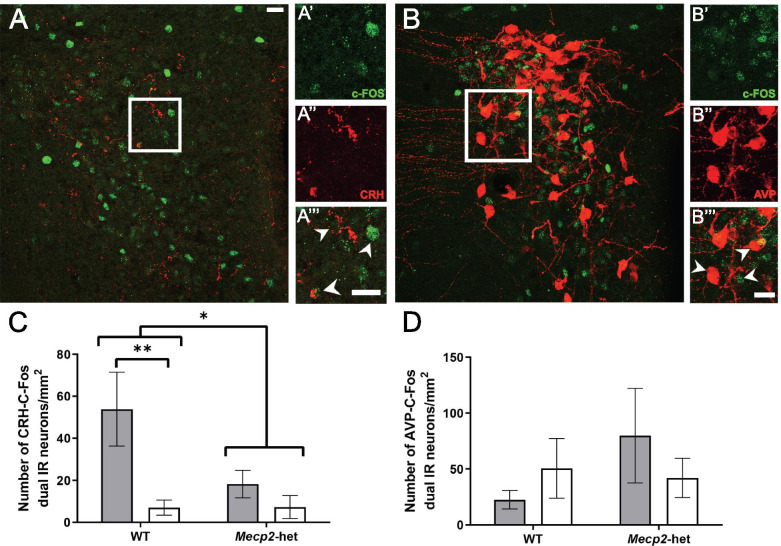
*Mecp2* haplodeficiency and maternal separation reduced the density c-FOS/CRH neuronal population in the paraventricular hypothalamic nucleus. Representative photomicrographs of **A** c-FOS/CRH and **B** c-FOS/AVP double immunofluorescence in the paraventricular hypothalamic nucleus (WT-naïve, *n* = 4; WT-MS, *n* = 7; *Mecp2*.het-naïve, *n* = 5; *Mecp2*.het-MS, *n* = 6). The number of c-FOS-positive cells that colocalize with CRH neurons was significantly reduced in Mecp2.het females and upon maternal separation. Quantification of the number of c-FOS neurons that colocalize with **C** CRH or **D** AVP. AVP, arginine vasopressin; c-FOS, FOS proto-oncogene; CRH, corticotropin-releasing hormone; *Mecp2*.het, *Mecp2* heterozygous females; MS, maternal separation; WT, wild type; vs, *versus*. *p < 0.05; **p < 0.01

Since the most significant effects of both *Mecp2* deficiency and MS in c-FOS-positive neurons were found in the Pa (Fig. 2C, D), we next asked whether the significant reduction in neuronal activation seen in WT-MS and *Mecp2*.het animals was due to a decrease in the activation of CRH neurons, AVP neurons, or both. To do so, we quantified the density of c-FOS-positive nuclei co-localizing with either CRH- or AVP-positive neurons (Fig. 3A–D). Data revealed a statistically significant genotype x group interaction in the number of c-FOS nuclei co-localized with CRH-positive neurons (*F*_(1, 17)_ = 4.920 *p* = 0.044; Fig. 3A, C). Post hoc tests showed that MS reduced the number of c-FOS/CRH double-labeled neurons in WT animals (Bonferroni correction, *F*_(1, 14)_ = 13.461 *p* = 0.003). Further, *Mecp2* deficiency per se resulted in a strong reduction of c-FOS/CRH double-labeled neurons (WT-naive vs *Mecp2*.het-naive, *F*_(1, 14)_ = 8.740 *p* = 0.010; *Mecp2*.het-naive vs *Mecp2*.het-MS, *p* > 0.5).

By contrast, no statistically significant effects of either factor were found for the density of c-FOS/AVP double-labeled neurons (Fig. 3B, D, *p* > 0.5). Previous studies suggested and increase in *Avp* expression levels after MS [6]. In the same line, although not reaching statistical significance, we found an average 60% increase in the density of AVP neurons in the Pa in WT-MS animals with respect to WT-naive mice (*p* = 0.055). On the other hand, the density of AVP cells in the Pa was not affected by *Mecp2* genotype (data not shown), in agreement with our previous study [36].

## Discussion

In this study, we used female mice with *Mecp2* haplodeficiency to study the interaction between MeCP2 and MS in anxiety-like behavior and in the functionality of underlying neuronal circuits. We found that, in WT females, MS induced a reduction in anxiety and a concomitant significant decrease in the activation of CRH neurons in the Pa, upon exposure to the EPM. Further, compared to WT littermates, adolescent *Mecp2*.het females show reduced anxiety and depressive-like behavior, accompanied by decreased activation of the CRH neuronal population in the Pa. Importantly, MS did not significantly affect any of those measures in *Mecp2* mutant females. Overall, our data suggests that a MeCP2-dependent mechanism is involved in controlling neuronal activity in stress-related circuits that are also responsive to ELS. And, importantly, a reduction of MeCP2 activity is sufficient to mimic MS effects.

### Mecp2 haplodeficiency recapitulates the effects of MS in anxiety-like behavior and in neuronal activation in the paraventricular nuclei

ELS can induce changes in gene expression, physiology (stress response) and behavior later in life. In the current study, we show that animals that were maternally separated (MS groups) display a reduction in the anxiety levels when tested in the bright EPM.

Previous studies demonstrated that MeCP2 is one of the factors mediating the effects of ELS in cognitive and emotional behaviors in mice [6]. Importantly, *Mecp2* haplodeficiency per se resulted in low levels of anxiety, mimicking the MS effects. In its first description, *Mecp2*.het females were reported as being asymptomatic at young ages, taking up to 6 months of age for 50% of females to develop overt symptoms [33]. Later though, we have shown that *Mecp2*.het pups have a delay in the development and acquisition of neurological reflexes [37], suggesting that *Mecp2* haplodeficiency at such young ages could affect the development of critical periods, and have an impact later in life. Here, we provide evidence that adolescent *Mecp2*.het females show indeed an altered sensitivity to early-life stress. In particular, at 6 weeks of age, *Mecp2*.het females present with less anxiety and less depressive-like behaviors, as compared to WT animals. Our data agrees with a previous study showing decreased anxiety in the EPM of 5 weeks old *Mecp2*.het females of mixed background [26]. More recently, Vogel Ciernia and colleagues [27] also reported increased time spent in the open arms of the EPM in 6–8 weeks old *Mecp2*.het females of the strain used in the present study. However, other studies using *Mecp2*-deficient models have reported conflicting results when assessing anxiety-like behavior.

To the reduction of the anxious phenotype of *Mecp2*.het females might contribute the genetic background of the dam by affecting maternal behavior, which in turn will affect pup’s response to a stressor [38, 39]. In this regard, *Mecp2*.het dams produce smaller litters and often neglect and cannibalize their offspring [40], in addition to impairments in maternal behavior [41]. In fact, studies using C57BL/6 animals (the background of our animal model) suggest that this strain shows resilience to MS, that could be attributed to the fact that these animals are poor breeders and dams [39, 42]. Altogether, cross-fostering studies to minimize the impact of maternal care on the offspring anxiety-like behaviors, ascribed to *Mecp2* genotype and/or strain, should be considered to clarify the effects here observed. Cross-fostering with CD-1 strain, which are good breeders, has actually proven beneficial in the evaluation of other RTT-like phenotypes [27]. Moreover, Lonetti and colleagues [43] showed that early life environmental enrichment, starting at PND10, rescued the anxious phenotype of young adult *Mecp2*^+/−^ females. These results warrant future studies disentangling the type and time windows of environmental interventions that could be beneficial to ameliorate RTT phenotypes.

A recent study in 6 months old, symptomatic, *Mecp2*.het questions that this phenotype is solely due to anxiety alterations. Strikingly, Flores Gutiérrez et al. [44] found that *Mecp2*.het females with trimmed whiskers did not show the anxiolytic phenotype in the EPM, suggesting that sensory hypersensitivity could be an important contributor of lowered anxiety. Nonetheless, our results showing decreased activation of the CRH neurons of the paraventricular nucleus indeed support a reduced anxiogenic response (see below). Taken together, these results reflect the complexity of assessing the causes of aberrant behaviors in both mouse models and RTT patients, given the sensory and motor impairments that characterize the disease. In the case of patients, this is further complicated by communication impairments. At the same time, our data supports the use of young *Mecp2*.het, largely pre-symptomatic females, as a valid model to study non-motor phenotype alterations.

EPM is an anxiogenic stressor and exposure to the maze induces neuronal activation in several brain areas associated with stress and anxiety [32, 45]. In our study, WT-MS animals showed a significant reduction in the number of c-FOS neurons specifically in the Pa and PV regions, as compared to naive animals, after EPM exposure. However, naive *Mecp2*.het females show low numbers of c-FOS neurons in the same regions, and MS has no additional effect. Our results show that MS precludes the activation of Pa and PV neurons, normally activated upon EPM exposure, and indicate that this may be a MeCP2-dependent process, as deficiency of *Mecp2* per se also precludes the activation of such neurons.

### Mecp2 haplodeficiency and MS preclude the activation of CRH neurons in the Pa

The Pa is a stress-responsive brain region and a key center of the HPA axis, containing CRH neurons that control adrenocorticotrophic hormone (ACTH) release from the hypophysis, which in turn activates the adrenal release of corticosterone to the bloodstream. ELS induces activity-regulated gene expression and alters the distribution of epigenetic marks, which MeCP2 reads, thereby modulating gene expression of a specific set of genes later in life [6, 7]. One of such genes is *Crh*, as rats subjected to MS showed reduced occupancy of several transcription factors, including MeCP2, in the promoter region of *Crh* gene accounting for its upregulation [23]. Our results show that both MS and MeCP2 deficiency specifically precluded the activation of CRH neurons in the Pa, concordant with the decreased anxiety phenotype exhibited by the animals. In line with our results, selective inactivation of *Mecp2* in hypothalamic neurons decreases *Crh* mRNA in the Pa [19]. Instead, the transgenic *Mecp2*^*308*^ mouse model, expressing a mutated form of MeCP2, presents with heightened anxiety and elevated levels of *Crh* expression in the hypothalamus [10, 20, 24]. The authors attributed this to the fact that mutated MeCP2 fails to occupy the proximal promoter of the *Crh* gene, which associates with elevated levels of *Crh* expression [10]. Indeed, in vitro studies with rat embryonic hypothalami showed that MeCP2 is necessary for maintaining basal levels of *Crh* gene expression, as MeCP2 knockdown leads to increased expression of *Crh* [46]. On the other hand, mice with double or triple levels of MeCP2 showed increased levels of *Crh* and displayed heightened anxiety-like behavior when subjected to the EPM and to the light-dark box [22]. The influence of strain background on behavior performance can be contributing to the apparently contradictory results of the different studies [26]. Nevertheless, we cannot exclude that zygosity, sex [20], or even *Mecp2* mutation type could also account for the apparently contradictory effects of MeCP2 on anxiety.

Our data showing reduced neuronal activation of CRH neurons, together with other studies showing a decrease [19] or increase [10, 20, 21, 46] of *Crh* expression in *Mecp2*-deficient models suggest that feedback loops ensuring the correct function of HPA axis are compromised in *Mecp2* mouse models. Consistent with this, modulation of the corticosteroid levels has proven beneficial in these models with a positive impact in locomotor/exploratory behavior, motor symptoms and lifespan [47, 48]. The analysis of serum (or plasma) corticosterone levels was not performed and stands as a caveat in this study. Nevertheless, our results of a decreased activation of CRH neurons in the Pa, upon exposure to an anxiogenic situation, provide indirect evidence that the levels of blood corticosterone are decreased or not altered in the *Mecp2*.het females. In line with our results, *Mecp2*^+/−^ females in a mixed background are less anxious and have an abnormal corticosterone response to stress, either with no differences or with decreased levels of corticosterone, when compared to control animals [26]. Moreover, Cosentino and colleagues [20], using a different RTT mouse model, showed that upon restrain stress Mecp^308^ females have lower levels of corticosterone than wild-type females.

Our data did not reveal a statistically significant effect of either MS or *Mecp2* deficiency in the activation of AVP neurons. Previous data showed that MS induced hypomethylation at the *Avp* enhancer region, and a low presence of bound MeCP2 in the same region, thereby increasing *Avp* expression specifically in the Pa [6]. Also, mice lacking fully functional MeCP2 (*Mecp2*^*308*^ null), but not mice with MeCP2 haplodeficiency (Mecp2^308^ het), show an increased expression of *Avp* in the hypothalamus [20]. In our study, although apparently higher compared to WT-naive, the high variability in the density of hypothalamic AVP neurons prevented us from drawing strong conclusions regarding the effect of MS. On the other hand, *Mecp2* deficiency had no effect in the density of AVP neurons in the Pa, in line with our previous results [36] and with the results obtained in mice with a selective inactivation of *Mecp2* in hypothalamic neurons [19].

Regarding the PV, this thalamic nucleus is a key hub of the circuits regulating emotional behaviors, and displays important connections with the hypothalamus, BST, and amygdala [49]. Indeed, its function is essential to regulate responses to stressors [50]. Thus, the decreased activation seen in the PV of *Mecp2*.het females might reflect, as in the case of Pa, an inability to appropriately respond to an anxiogenic situation. Finally, although we did not find a statistically significant effect in the BSTLD and LSV, these structures display a trend towards hypoactivation in both *Mecp2.*het and WT-MS females, similar to what was found in Pa and PV. This trend was not seen in the in the DG, suggesting that differences observed in c-FOS induction after EPM are specific of the emotional circuits.

### Early-life environment and *Mecp2* interact to modulate the risk of developing psychopathology in RTT and other psychiatric disorders.

*MECP2* constitutes a shared genetic risk factor to multiple neurodevelopmental disorders; primarily to RTT [11] and, to a lesser extent, to a range of other neurodevelopmental disorders including autism [51], schizophrenia [52], or intellectual disability [53] and to MDS [15]. Importantly, disturbances of MeCP2 during critical periods definition can constitute a risk factor for psychopathology in neurodevelopmental disorders, but also for anxiety- and stress-related disorders, by impairing the proper maturation of the HPA axis, which constitutes *per se* a risk factor for the development of psychopathology [2]. Indeed, long-time empirical reports (from clinicians and parents) suggest that anxiety is an important component of the behavioral phenotype of RTT. More recently, scientific studies confirmed disturbances in anxiety in patients with RTT [25], MDS [28], and in *Mecp2* mouse models [10, 22]. In the same line, Consentino and colleagues [24] showed that transgenic *Mecp2*^308/Y^ mice show susceptibility to develop PTSD-like symptomatology after a traumatic event, reinforcing the role of MeCP2 in psychopathology.

## Conclusions

Data presented in this study suggest that deficiency of MeCP2 early in development, through the regulation of the critical period of HPA axis, may underlie the alterations in anxiety behavior observed in adolescent *Mecp2*.het females. Future studies using models that allow a spatiotemporal control of MeCP2 expression should be used in order to prove its involvement and explore the underlying molecular mechanisms. It is unquestionable that changes in MeCP2 expression affect anxiety-like behaviors. However, further investigation is needed to fully elucidate the anxiety profile in RTT and *MECP2*-associated diseases. In addition, it is possible that there is currently an underreported association between *MECP2*, anxiety (panic disorder, phobias, generalized anxiety disorder) and stress-related disorders (posttraumatic stress disorder), and future studies in the field should screen for *MECP2* variants and also methylation levels (mCA and mCpG) of specific MeCP2 target genes (*CRH*, *AVP*, *BDNF*), which early in development are sensitive to the environment and modulate the response to stressors later in life.

In conclusion, this study provides evidence that a *Mecp2*-dependent mechanism is involved in controlling neuronal activity in stress-related circuits that are also responsive to early-life adversity. Moreover, data confirmed CRH pathway as a potential entry point for the treatment of anxiety in RTT and related conditions.

## Data Availability

All data generated or analyzed during this study are included in this published article.

## Abbreviations

ANOVA: Analysis of variance
AVP: Arginine vasopressin
BSTLD: Bed nucleus of the stria terminalis lateral division, dorsal part
c-FOS: FOS proto-oncogene
CRH: Corticotropin-releasing hormone
DAB: 3-3′-diaminobenzidine
DG: Dentate gyrus
ELS: Early-life stress
EPM: Elevated plus maze
FST: Forced swimming test
Het: Heterozygous
HPA: Hypothalamic-pituitary-adrenal
LSV: Lateral septum, ventral part
MDS: *MECP2* duplication syndrome
MeCP2: Methyl-CpG binding protein 2
*MECP2*: Methyl-CpG binding protein 2 gene
MS: Maternal separation
NDS: Normal donkey serum
NGS: Normal goat serum
OF: Open field
Pa: Paraventricular hypothalamic nucleus
PB: Phosphate buffer
PBS: Phosphate buffered saline
PND: Postnatal day
PV: Paraventricular thalamic nucleus
RT: Room temperature
RTT: Rett syndrome
TBS: Tris-buffered saline
Triton X100: Tx
WT: Wild-type

## References

[1] Bandelow B, Michaelis S. Epidemiology of anxiety disorders in the 21st century. Dialogues Clin Neurosci. 2015;17(3):327–35. 10.31887/DCNS.2015.17.3/bbandelow PMC4610617 26487813

[2] Craske MG, Stein MB, Eley TC, Milad MR, Holmes A, Rapee RM (2017). Anxiety disorders. Nat Rev Dis Primers.

[3] Guy J, Cheval H, Selfridge J, Bird A (2011). The role of MeCP2 in the brain. Annu Rev Cell Dev Biol.

[4] Chen WG, Chang Q, Lin Y, Meissner A, West AE, Griffith EC (2003). Derepression of BDNF transcription involves calcium-dependent phosphorylation of MeCP2. Science..

[5] Martinowich K, Hattori D, Wu H, Fouse S, He F, Hu Y (2003). DNA methylation-related chromatin remodeling in activity-dependent Bdnf gene regulation. Science.

[6] Murgatroyd C, Patchev AV, Wu Y, Micale V, Bockmühl Y, Fischer D (2009). Dynamic DNA methylation programs persistent adverse effects of early-life stress. Nat Neurosci.

[7] Stroud H, Su SC, Hrvatin S, Greben AW, Renthal W, Boxer LD (2017). Early-life gene expression in neurons modulates lasting epigenetic states. Cell..

[8] Picard N, Fagiolini M (2019). MeCP2: an epigenetic regulator of critical periods. Curr Opin Neurobiol.

[9] van Bodegom M, Homberg JR, Henckens MJAG. Modulation of the hypothalamic-pituitary-adrenal axis by early life stress exposure. Front Cell Neurosci. 2017;11:87. 10.3389/fncel.2017.00087 PMC5395581 28469557

[10] Mcgill BE, Bundle SF, Yaylaoglu MB, Carson JP, Thaller C, Zoghbi HY. Enhanced anxiety and stress-induced corticosterone release are associated with increased Crh expression in a mouse model of Rett syndrome. Proc Natl Acad Sci U S A. 2006;103(48):18267–72. 10.1073/pnas.0608702103 PMC1636379 17108082

[11] Amir RE, van den Veyver IB, Wan M, Tran CQ, Francke U, Zoghbi HY (1999). Rett syndrome is caused by mutations in X-linked MECP2, encoding methyl- CpG-binding protein 2. Nat Genet.

[12] Hagberg B (2002). Clinical manifestations and stages of Rett syndrome. Ment Retard Dev Disabil Res Rev.

[13] Santos M, Temudo T, Kay T, Carrilho I, Medeira A, Cabral H (2009). Mutations in the MECP2 gene are not a major cause of rett syndrome-like or related neurodevelopmental phenotype in male patients. J Child Neurol.

[14] Gonzales ML, LaSalle JM (2010). The role of MeCP2 in brain development and neurodevelopmental disorders. Curr Psychiatry Rep.

[15] Ramocki MB, Tavyev YJ, Peters SU (2010). The MECP2 duplication syndrome. Am J Med Genet A.

[16] Byiers BJ, Payen A, Feyma T, Panoskaltsis-Mortari A, Ehrhardt MJ, Symons FJ (2020). Associations among diurnal salivary cortisol patterns, medication use, and behavioral phenotype features in a community sample of rett syndrome. Am J Intellect Dev Disabilities.

[17] Peters SU, Byiers BJ, Symons FJ (2016). Diurnal salivary cortisol and regression status in MECP2 duplication syndrome. J Child Neurol.

[18] Lombardi LM, Baker SA, Zoghbi HY (2015). MECP2 disorders: from the clinic to mice and back. J Clin Investig.

[19] Fyffe SL, Neul JL, Samaco RC, Chao HT, Ben-Shachar S, Moretti P (2008). Deletion of Mecp2 in Sim1-expressing neurons reveals a critical role for MeCP2 in feeding behavior, Aggression, and the Response to Stress. Neuron.

[20] Cosentino L, Bellia F, Pavoncello N, Vigli D, D’Addario C, de Filippis B. Methyl-CpG binding protein 2 dysfunction provides stress vulnerability with sex- and zygosity-dependent outcomes. Eur J Neurosci. 2021. 10.1111/ejn.15165. 10.1111/ejn.15165 33655553

[21] Ren J, Ding X, Funk GD, Greer JJ (2012). Anxiety-related mechanisms of respiratory dysfunction in a mouse model of rett syndrome. J Neurosci.

[22] Samaco RC, Mandel-Brehm C, McGraw CM, Shaw CA, McGill BE, Zoghbi HY (2012). Crh and Oprm1 mediate anxiety-related behavior and social approach in a mouse model of MECP2 duplication syndrome. Nat Genet.

[23] Wang A, Nie W, Li H, Hou Y, Yu Z, Fan Q, et al. Epigenetic upregulation of corticotrophin-releasing hormone mediates postnatal maternal separation-induced memory deficiency. PLoS One. 2014;9(4):e94394. 10.1371/journal.pone.0094394 PMC3981802 24718660

[24] Cosentino L, Vigli D, Medici V, Flor H, Lucarelli M, Fuso A (2019). Methyl-CpG binding protein 2 functional alterations provide vulnerability to develop behavioral and molecular features of post-traumatic stress disorder in male mice. Neuropharmacology..

[25] Barnes KV, Coughlin FR, O’Leary HM, Bruck N, Bazin GA, Beinecke EB, et al. Anxiety-like behavior in Rett syndrome: Characteristics and assessment by anxiety scales. J Neurodev Disord. 2015;7(1):30. 10.1186/s11689-015-9127-4 PMC4571061 26379794

[26] Samaco RC, Mcgraw CM, Ward CS, Sun Y, Neul JL, Zoghbi HY. Female Mecp2+/- mice display robust behavioral deficits on two different genetic backgrounds providing a framework for pre-clinical studies. Hum Mol Genet 2013; 22(1):96–109. Available from: https://pubmed.ncbi.nlm.nih.gov/23026749/. [cited 2021 Feb 8] 10.1093/hmg/dds406 PMC3522402 23026749

[27] Vogel Ciernia A, Pride MC, Durbin-Johnson B, Noronha A, Chang A, Yasui DH (2017). Early motor phenotype detection in a female mouse model of Rett syndrome is improved by cross-fostering. Hum Mol Genet.

[28] Ramocki MB, Peters SU, Tavyev YJ, Zhang F, Carvalho CMB, Schaaf CP (2009). Autism and other neuropsychiatric symptoms are prevalent in individuals with MECP2 duplication syndrome. Ann Neurol.

[29] Macrí S, Mason GJ, Würbel H (2004). Dissociation in the effects of neonatal maternal separations on maternal care and the offspring’s HPA and fear responses in rats. Eur J Neurosci.

[30] Lehmann J, Feldon J. Long-term biobehavioral effects of maternal separation in the rat: Consistent or confusing? [Internet]. Vol. 11, Reviews in the Neurosciences. Freund Publishing House Ltd; 2000. p. 383–408. Available from: https://pubmed.ncbi.nlm.nih.gov/11065281/. [cited 2021 Feb 8] 10.1515/revneuro.2000.11.4.383 11065281

[31] Litvin Y, Tovote P, Pentkowski NS, Zeyda T, King LB, Vasconcellos AJ (2010). Maternal separation modulates short-term behavioral and physiological indices of the stress response. Horm Behav.

[32] Galvis-Alonso OY, Garcia AMB, Orejarena MJ, Lamprea MR, Botelho S, Conde CA (2010). A combined study of behavior and Fos expression in limbic structures after re-testing Wistar rats in the elevated plus-maze. Brain Res Bull.

[33] Guy J, Hendrich B, Holmes M, Martin JE, Bird A. A mouse Mecp2-null mutation causes neurological symptoms that mimic rett syndrome. Nat Genet. 2001;27(3):322–326. Available from: https://pubmed.ncbi.nlm.nih.gov/11242117/. [cited 2021 Feb 8] 10.1038/85899 11242117

[34] Franklin KBJ, Paxinos G (2008). The mouse brain in stereotaxic coordinates. 3rd ed.

[35] Schindelin J, Arganda-Carreras I, Frise E, Kaynig V, Longair M, Pietzsch T (2012). Fiji: An open-source platform for biological-image analysis. Nat Methods.

[36] Martínez-Rodríguez E, Martín-Sánchez A, Kul E, Bose A, Martínez-Martínez FJ, Stork O (2020). Male-specific features are reduced in Mecp2-null mice: analyses of vasopressinergic innervation, pheromone production and social behaviour. Brain Struct Funct.

[37] Santos M, Silva-Fernandes A, Oliveira P, Sousa N, Maciel P (2007). Evidence for abnormal early development in a mouse model of Rett syndrome. Genes Brain Behav.

[38] Feifel AJ, Shair HN, Schmauss C (2017). Lasting effects of early life stress in mice: interaction of maternal environment and infant genes. Genes Brain Behav.

[39] Own LS, Patel PD (2013). Maternal behavior and offspring resiliency to maternal separation in c57bl/6 mice. Horm Behav.

[40] Jugloff DGM, Logan R, Eubanks JH (2006). Breeding and maintenance of an Mecp2-deficient mouse model of Rett syndrome. J Neurosci Methods.

[41] Lau BYB, Krishnan K, Josh Huang Z, Shea SD (2020). Maternal experience-dependent cortical plasticity in mice is circuit- And stimulus-specific and requires MECP2. J Neurosci.

[42] Holmes A, Le Guisquet AM, Vogel E, Millstein RA, Leman S, Belzung C (2005). Early life genetic, epigenetic and environmental factors shaping emotionality in rodents. Neuroscience and Biobehavioral Reviews. Neurosci Biobehav Rev.

[43] Lonetti G, Angelucci A, Morando L, Boggio EM, Giustetto M, Pizzorusso T (2010). Early environmental enrichment moderates the behavioral and synaptic phenotype of MeCP2 null mice. Biol Psychiatry.

[44] Flores Gutiérrez J, de Felice C, Natali G, Leoncini S, Signorini C, Hayek J, et al. Protective role of mirtazapine in adult female Mecp2 +/-mice and patients with Rett syndrome. J Neurodev Disord. 2020;12(1) Available from: https://pubmed.ncbi.nlm.nih.gov/32988385/. [cited 2021 Feb 8]. 10.1186/s11689-020-09328-z PMC7523042 32988385

[45] Troakes C, Ingram CD (2009). Anxiety behaviour of the male rat on the elevated plus maze: Associated regional increase in c-fos mRNA expression and modulation by early maternal separation. Stress..

[46] Bhave SA, Uht RM (2017). CpG methylation and the methyl CpG binding protein 2 (MeCP2) are required for restraining corticotropin releasing hormone (CRH) gene expression. Mol Cell Endocrinol.

[47] Braun S, Kottwitz D, Nuber UA (2012). Pharmacological interference with the glucocorticoid system influences symptoms and lifespan in a mouse model of Rett syndrome. Hum Mol Genet.

[48] de Filippis B, Ricceri L, Fuso A, Laviola G (2013). Neonatal exposure to low dose corticosterone persistently modulates hippocampal mineralocorticoid receptor expression and improves locomotor/exploratory behaviour in a mouse model of Rett syndrome. Neuropharmacology.

[49] Kirouac GJ (2015). Placing the paraventricular nucleus of the thalamus within the brain circuits that control behavior. Neurosci Biobehav Rev.

[50] Hsu DT, Kirouac GJ, Zubieta JK, Bhatnagar S. Contributions of the paraventricular thalamic nucleus in the regulation of stress, motivation, and mood. Front Behav Neurosci. 2014;8:73. 10.3389/fnbeh.2014.00073 PMC3949320 24653686

[51] Coutinho AM, Oliveira G, Katz C, Feng J, Yan J, Yang C (2007). MECP2 coding sequence and 3′UTR variation in 172 unrelated autistic patients. Am J Med Genet Part B Neuropsychiatr Genet.

[52] Chen CH, Cheng MC, Huang A, Hu TM, Ping LY, Chang YS (2020). Detection of rare Methyl-CpG binding protein 2 gene missense mutations in patients with schizophrenia. Front Genet.

[53] Couvert P, Bienvenu T, Aquaviva C, Poirier K, Moraine C, Gendrot C, et al. MECP2 is highly mutated in X-linked mental retardation. Hum Mol Genet. 2001;10(9):941–6. 10.1093/hmg/10.9.941 11309367

